# Contribution of xpert MTB/RIF assay and Urine LF-LAM for the diagnosis of tuberculosis in children aged 5 – 14 years, at selected health facilities in Ethiopia, 2016 – 2019

**DOI:** 10.1371/journal.pone.0338557

**Published:** 2025-12-08

**Authors:** Muluwork Getahun, Yenew Kebede, Hilina Molalegn, Ayinalem Alemu, Getu Diriba, Waganeh Sinshaw, Mengistu Tadesse, Ephrem Tesfaye, Kassahun Belachew, Ameha Mekasha, Beniam Feleke

**Affiliations:** 1 Ethiopian Public Health Institute, Infectious Disease Research Directorate, Addis Ababa, Ethiopia; 2 Division of Laboratory Systems & Networks, Africa Centers for Disease Control and Prevention, Addis Ababa, Ethiopia; 3 University of Gondar comprehensive specialized Hospital, Addis Ababa, Ethiopia; 4 Black lion Hospital, Addis Ababa, Ethiopia; 5 Centers for Disease Control and Prevention, Division of Global HIV and TB, Addis Ababa, Ethiopias; Stellenbosch University, SOUTH AFRICA

## Abstract

**Background:**

Childhood tuberculosis (TB) remains under-reported and undiagnosed. A full complement of diagnostic tests is oftentimes unavailable in resource limited country like Ethiopia. This study assesses the contribution of Xpert MTB/RIF assay and urine LF-LAM for childhood TB diagnosis using sputum and urine samples.

**Method:**

A facility based cross-sectional study was conducted in children between 5 and 14 years of age. Sputum and urine samples were collected from children with presumptive TB. The samples were tested for TB using LF-LAM, Xpert MTB/RIF assay, concentrated smear microscopy, and culture. Diagnostic performance of Xpert MTB/RIF assay was analyzed and compared against culture, which was used as the gold standard. Urine LF-LAM test result was compared to a composite reference standard.

**Result:**

Of 576 participants with presumptive TB enrolled in the study, 519 (90.1%) had complete clinical data and bacteriological laboratory test results. Active TB was diagnosed in 14.1% (73/519), and bacteriological confirmation was made in 10.1% (52/515) of children with presumptive TB. The odds of being diagnosed with a bacteriologically confirmed TB are significantly higher in children who have household contact history with TB patient (aOR 2.27, P = 0.03) and age above 10 years (aOR 3.67, P < 0.001). Xpert MTB/RIF test had sensitivity of 79% using culture as the gold standard. Compared to smear microscopy, the sensitivity of the Xpert MTB/RIF assay increased by 50% for children aged 5–9 years and by 40% for children and adolescents living with HIV (C/ALHIV). All bacteriologically confirmed (n = 2) and clinically diagnosed TB children (n = 2) who live with HIV were tested positive for urine LF-LAM. The overall sensitivity of urine LF-LAM was 27.6% when using the composite reference standard, compared to 17.9% when the bacteriological reference standard was applied.

**Conclusions:**

Pulmonary TB diagnosis was greatly improved with the use of Xpert MTB/RIF assay, particularly in children aged 5–9 years and C/ALHIV who typically have difficulty producing good quality sputum. Urine LF-LAM performed well in children/adolescents who tested positive for HIV, but it performed poorly in the other variables, which suggests that urine LF-LAM testing did not play a critical role in TB diagnosis in children with negative HIV status.

## Introduction

Pulmonary tuberculosis (PTB) in children less than 15 years accounts for 11% of all tuberculosis (TB) globally [1]. Of the estimated global TB deaths, 14% were among children with human immunodeficiency virus (HIV) negative and 11% among children living with HIV (CLHIV). National TB programs in most countries notify less than half of the estimated TB in children. More than two third (69%) of childhood TB under the age of 5 years, and 40% of childhood TB remain unreported and undiagnosed [2]. In Ethiopia, childhood TB accounts for 10% of all forms of TB [1]. However, only less than half of the estimated TB in children is notified by the National TB Program, of which 28% are between the ages of 0 and 4 years; the remaining 72% are between the ages of 5 and 14 years. In 2022, 33% of TB in children notified by the national TB Program were bacteriologically confirmed PTB, 28% were clinically diagnosed PTB, and 38% were extra-pulmonary tuberculosis (EPTB) [3]. Generally, microbiological confirmation was only possible for 30% of children with pulmonary TB [4]. Therefore, the true burden of TB is underestimated [2].

Childhood TB diagnosis relies on a thorough assessment of all evidence derived from a careful history, clinical examination, relevant investigations, including radiologic and microbiologic evaluations [5]. Diagnosis of childhood TB is usually challenging due to its non-specific symptoms, difficulty in obtaining respiratory samples, and limited access to sensitive diagnostic methods [5,6]. To confirm childhood TB, different samples and sensitive diagnostic methods are frequently sought after. The Xpert MTB/RIF (Cepheid, USA) test, increases *Mycobacterium tuberculosis* (MTB) detection and genetic mutations associated with resistance to rifampicin, thereby aiding in the selection of treatment choices [7]. The urine lateral flow lipoarabinomannan (LF-LAM) (Determine Alere, Abbott, USA) antigen tests assists in the diagnosis of TB patients co-infected with HIV in conjunction with WHO recommended test such as Xpert MTB/RIF [8,9].

Children also frequently have negative smear results for acid fast bacilli because of their difficulty expectorating sputum and their reduced bacillary load [10]. Diagnosis of TB in children using non-respiratory samples for those patients unable to expectorate sputum is an invaluable means to confirm pediatric TB [10,11]. Soluble LAM is produced in both pulmonary and extra-pulmonary TB disease and eliminated in urine, making it a promising biomarker for TB, especially in children who are frequently missed by sputum-based diagnosis [12,13]. Therefore, the present study is designed to assess the contribution of Xpert MTB/RIF and urine LF-LAM to childhood TB diagnosis using urine and sputum samples.

## Methods

### Study population, design, and participant enrollment

This is a facility-based cross-sectional study conducted at 20 health facilities in Addis Ababa, Oromia, Amhara, and former Southern Nations, Nationalities and Peoples (SNNPR) regions of Ethiopia. The study enrolled children who visited the health facilities from June 2016 through February 2019. The sites were purposively selected based on pediatric TB patient load and ease of sample transport and monitoring. Children who seek care at those selected health facilities were targeted for the study. All participants visiting the study sites pass through standard triage protocols, which include administering the TB four-symptom screen. Consecutive participants identified to have presumptive TB by clinicians or physicians (who are also focal persons of the study) and who were able to provide sputum/urine samples were invited to participate in the study. Demographic and clinical data were collected by trained study focal persons using standardized tools developed for the study. Additionally, we collect data on history of TB contact, and chest x-ray findings (S1 Table). History of contact is defined as an exposure to TB through household contact with index TB patient in the preceding 2 years [14,15]. Information on the history of contact was obtained from their parent/guardian. This study was reviewed and approved by the Ethiopian Public Health Institute ethical review board.

### Sample size determination

The sample size was calculated using the equation n>= (Z)2p (1-p)/x2, where n = sample size; Z is the desired confidence level, p is the sensitivity of Xpert MTB/RIF and LF_LAM, and x is the margin of error. Since the sensitivity of Xpert MTB/RIF and LF_LAM in children has not been evaluated in our country; thus, we take 50% sensitivity, 95% CI, a margin of error of 0.05, and with a 1.5 design effect. The total sample size was 576.

### Laboratory test

After enrollment, morning urine and sputum samples were collected from the participants with presumptive TB and transported (maintaining cold chain) to the national TB reference laboratory at the Ethiopian Public Health Institute (EPHI) for further TB diagnostic tests. Urine LF-LAM (Determine Alere, Abbott, USA) was done on the spot at respective study health facilities as per the manufacture’s recommendation [16]. Xpert MTB/RIF assay and MGIT culture were done at EPHI on urine and sputum samples. The sputum and the urine samples were split into two; the first part (at least 2 ml) was allocated for the Xpert MTB/RIF test, and the second part was used for the MGIT culture. Additionally, the urine sample was concentrated by centrifuging at 3000 g for 15 minutes. Xpert MTB/RIF assay (Cepheid, USA) test was done on the first half of raw sputum samples, unprocessed and concentrated urine samples [17]. The second half of the raw sputum sample and concentrated urine were processed with N-acetyl-L-cysteine-sodium hydroxide (NaOH-NALC) for the MGIT culture. Concentrated smear microscopy examination was done using Ziehl-Neelsen (ZN) on sputum sediment. Positive MGIT cultures were checked for contamination with ZN and blood heart infusion (BHI) agar [18]. Identification of *M. tuberculosis complex* from other mycobacterium species was done on positive MGIT culture using TB Ag MPT64 (SD bioline, Germany) [19]. Negative culture results were issued after 42 days. The site was notified if any of the bacteriological tests (other than Urine LAM test) were positive for MTBC.

### Operational definition

Children with presumptive TB were categorized by measures of clinical disease state of PTB, i.e., bacteriologically confirmed, probable, possible, unlikely, and not tuberculosis (S2 Table) [5,6]. The classification of disease certainty was done after we obtained all the required data, including the MGIT culture result. A bacteriologically confirmed TB is a person from whom biological specimen is positive by smear microscopy, Xpert MTB/RIF or culture [10]. Clinically diagnosed TB patients are individuals who received anti-TB treatment based on their clinicians’ decision (using other clinical parameters to establish diagnosis) while having negative laboratory test results.

### Data analysis

Data were analyzed using SPSS. The frequency and percentage of PTB diagnosed by bacteriological and/or clinical evaluation were further compared by clinical and demographic variables. Furthermore, bivariable and multivariable logistic regression models were used to assess the relationship between PTB and various demographic and clinical factors. The variables that had a p-value of ≤0.2 from the bivariable analysis were included in the multivariable logistic regression models. Sensitivity, specificity, and negative and positive predictive values of smear microscopy (on concentrated sputum sample) and Xpert MTB/RIF were compared with the MGIT sputum culture result. Proportions were used to describe bacteriologically confirmed TB patient using urine samples. The diagnostic contributions of urine LF-LAM were assessed by clinical diagnostic group, bacteriological test results, and anti-TB treatment. The diagnostic performance of urine LF-LAM was compared with bacteriological tests (smear, Xpert MTB/RIF, and culture). Then the analysis was further stratified by HIV status and history of TB contact information. P-values of less than 0.05 were statistically significant.

## Results

**Fig 1:**
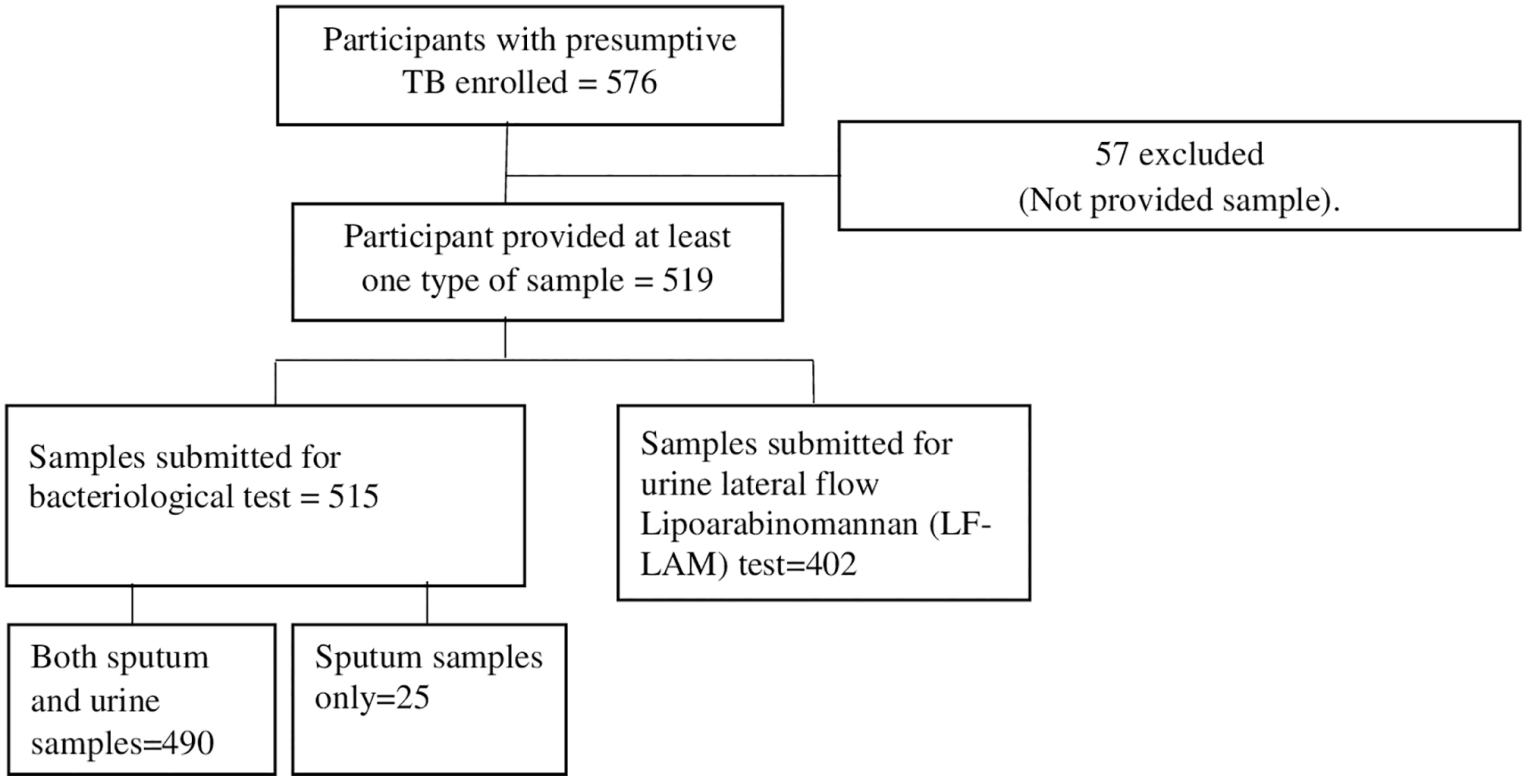
Presumptive TB participant enrolled, and samples collected from 2016-2019 in Ethiopia.

A total of 576 participants with presumptive PTB were enrolled in the study (Fig 1), with specimens collected from 519 (90.1%), and 515 participants had bacteriological test results. Age, sex, history of household TB contact, and HIV test result were reported for 91.8%, 92.4%, 74.7% and 51% of the participants having bacteriological test results. Males made up 50.4% of the study participants. The median age of participants was 10 years (interquartile range: 7 to 12). About half (56%) of the participants were older than 10 years. HIV test results were available for 51% of the participants, and out of those for whom HIV test results were available, 12.5% were HIV-positive. In 22.3% of the participants, parents/guardians reported a history of household contact with index pulmonary TB in the last 2 years.

Following the clinical and bacteriological test evaluation, a total of 73/519 (14.1%) participants were diagnosed with TB. Of these, 10.1% (52/515) were bacteriologically confirmed, and the remaining 4% (21/519) were clinically diagnosed. PTB was present in 21.2% of C/ALHIV and 23.0% of children and adolescents with a contact history of TB (Table 1). The odds of being diagnosed with bacteriologically confirmed TB for children aged 10–14 are 3.67 times higher than the odds of being diagnosed with a bacteriologically confirmed case of TB for children aged 5–9 years. Similarly, the odds of being diagnosed with a bacteriologically confirmed TB for children who’ve had contact with a person with active TB in the past two years are 2.27 times higher than the odds of being diagnosed with a bacteriologically confirmed TB for children who did not have contact with a person with active TB (Table 2).

**Table 1:** Bivariable logistic regression analysis of pulmonary TB by age group, sex, contact history and HIV test result.

Case classification	Variables		Total	N	%		
						OR[95% CI]	P-value
Clinically and bacteriologically diagnosed	Age Group(years)	5-9	209	17	8.1	R	
		10-14	267	54	20.2	2.863[1.605-5.108]	<0.001
		Not reported	43	2	4.8	0.551[0.123-2.478]	0.437
	Sex	Female	236	30	12.7	R	
		Male	243	41	16.6	1.394[0.837-2.319]	0.201
		Not reported	40	2	5	0.361[0.083-1.576]	0.176
	Contact history within 2yr	No	301	37	12.3	R	
		Yes	87	20	23.0	2.130[1.161-3.906]	0.015
		Unknown	131	16	12.2	0.993[0.531-1.856]	0.982
	HIV test results	Negative	230	27	11.7	R	
		Positive	33	7	21.2	2.024[0.802-5.111]	0.136
		Unknown	256	39	15.2	1.351[0.798-2.288]	0.263
Bacteriologically diagnosed	Age Group years	5-9	208	10	4.8	R	
		10-14	265	40	15.1	3.520[1.715-7.223]	0.001
		Not reported	42	2	4.8	0.990[0.209-4.691]	0.990
	Sex	Female	236	24	10.2	R	
		Male	240	26	10.8	1.073[0.597-1.929]	0.813
		Not reported	39	2	5.1	0.477[0.108-2.106]	0.329
	Contact within 2yr	No	299	23	7.7	R	
		Yes	86	13	15.1	2.137[1.033-4.423]	0.041
		Unknown	130	16	12.3	1.684[0.858-3.306]	0.130
	HIV status	Negative	230	21	9.1	R	
		Positive	33	5	15.2	1.777[0.621-5.089]	0.284
		Unknown	252	26	10.3	1.145[0.625-2.097]	0.661

R: Reference.

**Table 2:** Multivariable logistic regression analysis of pulmonary TB by age group and contact history.

Variables		Multivariable OR[95% CI]	P-value
Age Group years	5-9	R	
	10-14	3.670[1.779-7.573]	<0.001
	Not reported	0.637[0.125-3.248]	0.588
Contact within 2yr	No	R	
	Yes	2.137[1.033-4.423]	0.03
	Unknown	1.684[0.858-3.306]	0.021

Out of 52 bacteriologically confirmed TB patients, 57.7%, 75% and 77% had samples that were positive for smear microscopy (on concentered sputum sample), Xpert MTB/RIF assay, and culture, respectively (Table 3). The number of bacteriologically positive patients by sample types and methods is presented in S1 Fig. Performance characteristics of sputum smear microscopy and Xpert MTB/RIF are summarized in Table 4. Smear microscopy and Xpert MTB/RIF had an overall sensitivity of 60% and 79%, respectively, when using culture as the gold standard. Compared to smear microscopy, the Xpert MTB/RIF assay had higher sensitivity across all categories. A notable increase in sensitivity was observed among C/ALHIV (40%) and children aged 5–9 years old (50%).

**Table 3:** Number of bacteriological confirmed TB by sample types and diagnostic methods.

Sample type	Total tested	Positive	Smear Microscopy		Xpert MTB/RIF assay		MGIT Culture	
			Tested	Positive	Tested	Positive	Tested	Positive
Total	515	52	503	30	510	39	469	40
Sputum	515	51	503	30	485	37	435	40
Urine	490	8	NA	NA	484	6	403	3

NA: not applicable.

**Table 4:** Performance characteristics of sputum smear microscopy and Xpert MTB/RIF compared to gold standard by demographic and clinical variables.

Test method	Variables	Sensitivity	Specificity	PPV	NPV	Accuracy
Xpert MTB/RIF assay	Overall	78.9	98.7	85.7	97.9	96.8
	Age 5–9yrs	66.7	99.4	80	98.7	98.2
	Age 10–14yrs	80	97.8	85.7	96.8	95.3
	HIV-Positive	100	100	100	100	100
	HIV-Negative	82.4	98.8	87.5	98.1	97.2
	Contact within 2yr	81.8	100	100	96.9	97.3
	No known contact in 2yr	80	98.1	75	98.6	96.9
Smear microscopy	Overall	60	98.7	82.8	96	95.1
	Age 5–9yrs	16.7	98.8	33.3	97	95.8
	Age10–14yrs	65.6	98.4	87.5	94.5	93.8
	HIV-Positive	60	100	100	90.9	92
	HIV-Negative	52.9	98.8	81.8	95.5	94.7
	Contact within 2yr	63.6	100	100	94.1	94.7
	No known contact in 2yr	62.5	98.6	76.9	97.3	96.2

Urine LF-LAM assay results were available for 402 individuals; the remaining 88 patients did not have LF-LAM results due to a test kit shortage. Urine LF-LAM was positive for 10.7% (43/402) of the participants. Urine LF-LAM positivity rates were 17.9%, 23.1%, and 16.9% for confirmed, probable, and possible TB, respectively, while it was 4.7% in participants who were classified as ‘not having TB’. Among those children bacteriologically negative but clinically diagnosed with TB, 9 out of 10 children were tested positive for urine LF-LAM (Table 5). The performance characteristics of urine LF-LAM are shown in Table 6. The overall sensitivity of urine LF-LAM was 27.6% and its specificity was 92.2% when using the composite reference standard. In contrast, when applying the bacteriological reference standard, the sensitivity decreased to 17.9% and the specificity was 90%. Even though the number is too small, all bacteriologically confirmed (n = 2) and clinically diagnosed TB (n = 2) children who live with HIV have positive urine LF-LAM test results.

**Table 5:** The proportion of urine LF-LAM positivity by clinical diagnostic group, bacteriological test results and anti-TB treatment.

Classification		Total	LF-LAM positive	Positivity Rate
Clinical Diagnostic Groups	Overall	402	43	10.7
	Confirmed	39	7	17.9
	Probable	13	3	23.1
	Possible	83	14	16.9
	TB Unlikely	105	11	10.5
	Not evaluated	12	1	8.3
Bacteriologically test result and anti-TB treatment	Bacteriologically positive and treated	39	7	17.9
	Bacteriologically negative and treated	10	9	90
	Bacteriologically negative and not treated	341	27	7.9
	Bacteriologically NA and not treated	3	3	100

NA: not available.

**Table 6:** Performance characteristic of urine LF-LAM test compared with bacteriological diagnosis.

Gold standard	Variables	Total	Sensitivity	Specificity	PPV	NPV	Accuracy
Clinical and bacteriological diagnosis	Overall	399	27.6	92.2	37.2	88.3	82.8
	HIV-positive	26	100	86.4	57.1	100	88.5
	HIV negative	201	21.7	94.9	35.7	90.4	86.6
	Contact within 2 yr.	74	41.2	87.7	50.0	83.3	77.3
	No known contact in 2yr	259	27.6	93.1	33.3	91.1	85.8
Bacteriological diagnosis	Overall	399	17.9	90	16.3	91	83
	HIV positive	26	100	79.2	28.6	100	80.8
	HIV negative	201	17.6	94.0	21.4	92.5	8.6
	Contact within 2 yr.	74	30.0	82.8	21.4	88.3	75.
	No known contact in 2yr	259	17.6	91.3	12.8	94.0	86.5

## Discussion

In order to identify optimal approaches for the diagnosis of TB in children, the current study used clinical and laboratory data, including different diagnostic tests using urine and sputum samples. Our result showed that the use of bacteriological tests, including molecular tests, in children with presumptive TB, increases TB diagnosis [20,21]. We found that the odds of being diagnosed with a bacteriologically confirmed TB are higher for children who had a history of TB contact than their counterparts, indicating the importance of meticulously assessing contact history with TB patient(s) in this age group [22]. Compared to smear microscopy, a notable increase in TB detection using the Xpert MTB/RIF assay was observed among children aged 5–9 years and C/ALHIV, which suggests that these groups of clients who typically have difficulty producing good-quality sputum benefit the most from Xpert MTB/RIF detection. Urine LF-LAM performed well (sensitivity = 100%) in children/adolescents who tested positive for HIV, but it performed poorly (sensitivity = 17.6–30%),in the other variables, which suggests that urine LF-LAM testing did not play a critical role in TB diagnosis in children with negative HIV status.

Our study showed a higher proportion (10.1%) of bacteriologically confirmed TB compared to previous studies (4.2–7.1%) [23,24]. The difference may be due in part to the use of MGIT culture in addition to the Xpert MTB/RIF assay. Sensitive diagnostics, such as the Xpert MTB/RIF assay and MGIT culture, may not be widely available in clinical settings. In addition to the standard TB symptom screening criteria, children with chest radiographic abnormalities who were able to provide a sample were included in our study. This may have increased the TB screening yield, resulting in the inclusion of more children likely to have TB [5,6].

Children play an important role in the epidemiology of tuberculosis since they serve as a marker of recent transmission [10,14]. In this study, we found that the odds of being diagnosed with a bacteriologically confirmed TB are 2.27 times higher for children who had contact with a person(s) with active TB than for children who had no contact with an active TB. Similar studies have found that children with a history of TB contact were at greater risk of developing tuberculosis [25,26]. In a facility and community-based research conducted in Ethiopia, bacteriologically confirmed PTB was found in 20.5% and 6.9% of children with a history of TB contact, respectively [14,27]. The yield of bacteriological confirmation is higher in children who have a history of TB contact with TB patients. This implies that it is crucial to assess the contact history of these children during routine evaluations at health care facilities [22].

Lack of sensitive point-of-care tests is one of the challenges that hinders the timely detection and management of TB in children [10]. Our study showed the sensitivity of the Xpert MTB/Rif assay was 79% which is much higher than findings from other studies that showed sensitivity values in the range of 45% − 54% [28–30]. This could be due to differences in the target population; our study excluded children under 5 years old [10,14].

Xpert MTB/RIF assay diagnostic performance varied by children’s age and HIV test results. We found a significant difference in the odds of being diagnosed with a bacteriologically confirmed TB patient between children aged 10–14 years (aOR 3.6, P = 0.001) and those aged 5–9 years. Moreover, the Xpert MTB/RIF assay showed a sensitivity of 80% in the older age group compared to 66.7% in the younger age group. This finding was consistent with earlier studies conducted in Nigeria and China [31,32]*.*Conversely, relative to smear microscopy, a notable increase in TB detection using the Xpert MTB/RIF assay was observed among younger children and C/ALHIV. Our results indicated that the Xpert MTB/RIF assay was 50% more sensitive than smear microscopy for children aged 5–9 years and 40% more sensitive for C/ALHIV. Similarly, studies demonstrated a significantly higher detection rate in younger children and among C/ALHIV than smear microscopy [33–36], indicating that these groups benefit the most from Xpert MTB/RIF detection. The Xpert MTB/RIF assay improves the diagnostic yield in children who might be missed by microscopy due to low bacillary loads or inability to produce good-quality sputum.

The WHO strongly recommends using urine LF-LAM to assist in the diagnosis of TB disease in adults and C/ALHIV [7]. In our study, all bacteriologically confirmed (n = 2) and clinically diagnosed TB children (n = 2) who live with HIV were tested positive for urine LF-LAM. Our findings were comparable with studies conducted in South Africa and Uganda [37–39]. In contrast, our study found that the overall sensitivity of urine LF-LAM was 27.6% when using the composite reference standard. However, when the bacteriological reference standard was applied, the sensitivity decreased to 17.9%.. Variability in diagnostic performance has been documented among bacteriologically confirmed and clinically diagnosed TB [12,39,40]. This variation could be explained by differences in the concentration of soluble LAM found in urine specimens and the specificity of LF-LAM antibody used for detection, which may limit its diagnostic performance [12,13]. The potential contribution of the urine LF-LAM test for childhood TB diagnosis is demonstrated by the sensitivity difference when utilizing both bacteriological and clinical reference standards for active TB diagnosis. These findings warrant further investigation.

### Limitations of the study

The study sites were purposely selected based on patient load; therefore, the data obtained from these sites may not precisely represent the childhood TB burden in Ethiopia. We have missing data on age, sex, HIV status, and history of TB contact, which might lead to overestimation or underestimation of the proportion of TB patients in each category. The study excluded children under the age of five years due to anticipated difficulties in obtaining sputum samples, and gastric aspiration was not a standard method for obtaining samples at the study sites. As a result, the findings may not apply to children of all ages. Clinically diagnosed TB patients were determined using information obtained at the initial visit. Those children who did not have a documented clinical response might be reclassified as having TB. Therefore, clinically diagnosed TB in this study does not account for the follow-up tests performed on clients whose TB was not detected at baseline but identified during follow-up. Radiological result interpretation may vary across sites which likely influence the “probable” or the “possible” TB classification.The contribution of urine sample for the diagnosis of TB was only studied in children/adolescents with presumptive PTB, so the findings may not be generalizable to children having presumptive EPTB.

## Conclusions

This study highlights the importance of implementing enhanced TB finding approaches that include the use of rapid and sensitive diagnostic tools such as Xpert MTB/RIF. Expanding access to Xpert MTB/RIF assay diagnostic services would help to enhance prompt TB detection in children, which is critical for timely treatment and better outcomes. Children under the age of 10 years with presumptive TB who frequently have difficulty in expectorating sputum benefit the most from Xpert MTB/RIF testing so consistent use of GeneXpert testing in children with presumptive TB likely improves TB detection in this age group. Urine LF-LAM performed well in children/adolescents who tested positive for HIV; however, it performed poorly regarding other variables. This indicates that urine LF-LAM testing did not significantly contribute to TB diagnosis in children with negative HIV status.

## Supporting information

S1 Table Criteria for patient enrollment. (DOCX)

S2 Table Clinical definition categories for tuberculosis in children. (DOCX)

S1 Fig The number of bacteriologically positive patients by sample type and methods. A) Bacteriologically positive patients using sputum samples; B) Bacteriologically positive patients using sputum and urine samples. (TIF)
