# Supplementary material for: Contribution of xpert MTB/RIF assay and Urine LF-LAM for the diagnosis of tuberculosis in children aged 5 – 14 years, at selected health facilities in Ethiopia, 2016 – 2019
Source: PLoS One. 2025 Dec 8;20(12):e0338557. doi: 10.1371/journal.pone.0338557 (PMC12685164; doi:10.1371/journal.pone.0338557)
Supplement: S1 Table — (DOCX) [file pone.0338557.s001.docx]

Supplementary table 1: Criteria for patient enrollment

| Criteria | Definition | Observation |
| --- | --- | --- |
| Tuberculosis exposure | History of exposure to *M. tuberculosis*. Reported exposure to tuberculosis patient (close household contact) within the preceding 24 months | Confirmed contact (smear positive and/or culture positive, or tuberculosis treatment). OR  Verbal report (smear positive and/or culture positive, or tuberculosis treatment). |
| Clinical Symptoms and Signs Suggestive of Tuberculosis | Persistent cough | >2 weeks |
|  | Fever reported by a guardian or objectively recorded at least once. | Persistent (>1 week) and unexplained fever (>38^0^C) |
|  | lethargy or reduced playfulness reported by the guardian | Persistent, unexplained lethargy or decrease in Playfulness/activity. |
|  | Weight loss/failure to thrive | Unexplained weight loss: 5% reduction in weight compared with the highest weight recorded in last 3 months.  Significant weight loss perceived by the parent/guardian in the last 3 months  Failure to thrive and  Not responding to nutritional rehabilitation |
| Radiological Suggestive | Interpreted by radiologist and/or physician | Classify as suggestive of TB or not suggestive of TB |
