# Supplementary material for: Contribution of xpert MTB/RIF assay and Urine LF-LAM for the diagnosis of tuberculosis in children aged 5 – 14 years, at selected health facilities in Ethiopia, 2016 – 2019
Source: PLoS One. 2025 Dec 8;20(12):e0338557. doi: 10.1371/journal.pone.0338557 (PMC12685164; doi:10.1371/journal.pone.0338557)
Supplement: S2 Table — (DOCX) [file pone.0338557.s002.docx]

Supplementary table 2: Clinical definition categories for tuberculosis in children

| Clinical Diagnostic Groups | Definition of Categories |
| --- | --- |
| Confirmed tuberculosis | At least 1 of the signs and symptoms suggestive of tuberculosis **and** |
|  | Bacteriologically confirmation |
| Probable tuberculosis | At least 1 of the signs and symptoms suggestive of tuberculosis **and** |
|  | Chest radiography is consistent within intrathoracic disease due to  Mycobacterium tuberculosis **and** |
|  | There is at least one of the following:   1. A positive clinical response to anti-tuberculosis treatment 2. Documented exposure to M. tuberculosis |
| Possible tuberculosis | At least 1 of the signs and symptoms suggestive of tuberculosis **and** |
|  | Either one of the following:   1. A positive clinical response to anti-tuberculosis treatment 2. Documented exposure to M. tuberculosis or   chest radiography is consistent within intrathoracic disease due to  *Mycobacterium tuberculosis* |
| Tuberculosis unlikely | Symptomatic but not fitting the above definitions and no alternative diagnosis established |
| Not tuberculosis | Fitting the diagnosis for tuberculosis unlikely but with an established alternative diagnosis |
