# Supplementary figures and images for: Contribution of xpert MTB/RIF assay and Urine LF-LAM for the diagnosis of tuberculosis in children aged 5 – 14 years, at selected health facilities in Ethiopia, 2016 – 2019
Source: PLoS One. 2025 Dec 8;20(12):e0338557. doi: 10.1371/journal.pone.0338557 (PMC12685164; doi:10.1371/journal.pone.0338557)

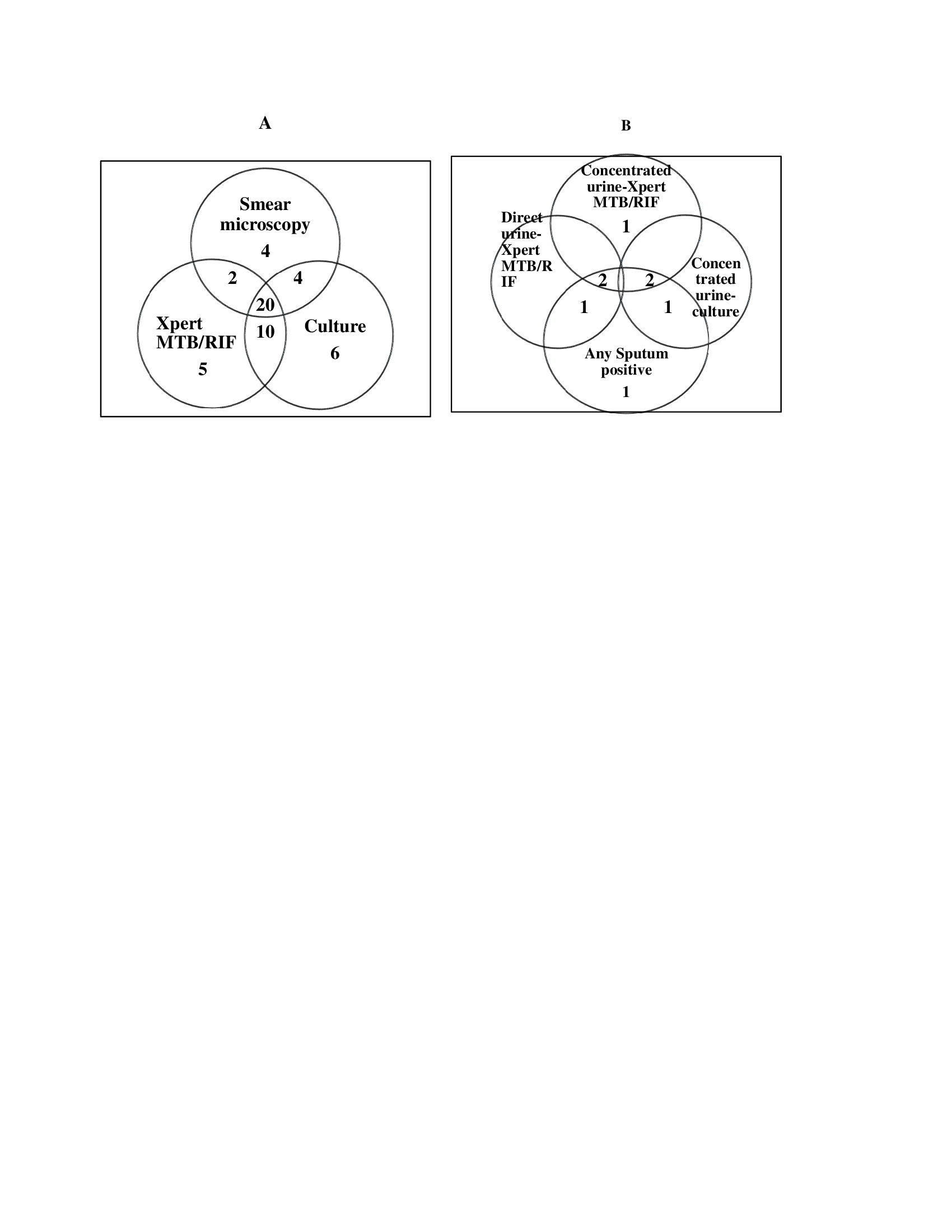

Supplement: S1 Fig — (TIF) [file pone.0338557.s003.tiff]
